# Impact of Spectral Notch Width on Neurophysiological Plasticity and Clinical Effectiveness of the Tailor-Made Notched Music Training

**DOI:** 10.1371/journal.pone.0138595

**Published:** 2015-09-25

**Authors:** Robert Wunderlich, Pia Lau, Alwina Stein, Alva Engell, Andreas Wollbrink, Claudia Rudack, Christo Pantev

**Affiliations:** 1 Institute for Biomagnetism and Biosignalanalysis, University Hospital of Münster, Münster, Germany; 2 Institute for Physiological Psychology, University of Bielefeld, Bielefeld, Germany; 3 Department of Otolaryngology, University Hospital of Münster, Münster, Germany; Kyoto University, JAPAN

## Abstract

Tinnitus, the ringing in the ears that is unrelated to any external source, causes a significant loss in quality of life, involving sleep disturbance and depression for 1 to 3% of the general population. While in the first place tinnitus may be triggered by damage to the inner ear cells, the neural generators of subjective tinnitus are located in central regions of the nervous system. A loss of lateral inhibition, tonotopical reorganization and a gain-increase in response to the sensory deprivation result in hypersensitivity and hyperactivity in certain regions of the auditory cortex. In the tailor-made notched music training (TMNMT) patients listen to music from which the frequency spectrum of the tinnitus has been removed. This evokes strong lateral inhibition from neurons tuned to adjacent frequencies onto the neurons involved in the tinnitus percept. A reduction of tinnitus loudness and tinnitus-related neural activity was achieved with TMNMT in previous studies. As the effect of lateral inhibition depends on the bandwidth of the notch, in the current study we altered the notch width to find the most effective notch width for TMNMT. We compared 1-octave notch width with ½-octave and ¼-octave. Participants chose their favorite music for the training that included three month of two hours daily listening. The outcome was measured by means of standardized questionnaires and magnetoencephalography. We found a general reduction of tinnitus distress in all administered tinnitus questionnaires after the training. Additionally, tinnitus-related neural activity was reduced after the training. Nevertheless, notch width did not have an influence on the behavioral or neural effects of TMNMT. This could be due to a non-linear resolution of lateral inhibition in high frequencies.

## Introduction

Tinnitus is known as the perception of a sound without any external source. For most people transient tinnitus is common, yet for 10 to 15% of the general population tinnitus remains an ongoing condition [1]. While many people adapt to their tinnitus, for 1 to 3% tinnitus means a significant reduction of quality of life [2]. Common complaints are sleeping problems, stress and even depression. The risk to experience tinnitus increases with increasing age [3]. Since our population is growing older, it is realistic to expect that the percentage of people with tinnitus will even increase in the future. However, also young people are affected by tinnitus, e.g. the widespread and often very loud use of mobile music players with insert earphones during recreational time is related to an increase of hearing problems and tinnitus [4,5]. Tinnitus is not only causing a decreased quality of life for some patients, but is also a health issue with economic effects [6]. Considering this, it is consequent that the scientific and medical society puts much effort into developing efficient treatments against tinnitus.

Unspecific treatments have attempted to facilitate blood circulation in the cochlea via infusion, medication or a hyperbaric environment to enable regeneration of affected hair cells. While this might help during acute tinnitus that is for instance caused by noise traumas and cochlea insults, it is not an effective treatment for chronic tinnitus [7]. Yet, our understanding of the neurophysiological fundamentals of tinnitus has improved, which enables to target tinnitus in more specific ways. Tinnitus is assumed to be the result of maladaptive plasticity in the auditory cortex in reaction to the degeneration of neurons in the cochlea [8]. In normal hearing, excitation does not only spread to higher neuronal levels but adjacent neurons also inhibit each other, a phenomenon called lateral inhibition [9,10]. When hearing loss occurs, neurons in the auditory cortex do not reduce or even only lose their afferent input from the cochlea. Moreover, the lateral inhibition affecting neurons coding neighboring frequencies also decreases [11,12]. Tonotopical reorganization sets in and neurons with lost input start responding to input from non-affected frequency areas, preferably at the audiometric edge frequency [13]. Together with the lost inhibition this results in an over-representation of frequency regions at the edge of the hearing loss [8]. The overall loss of input from sensory cells might also change the sensitivity functions of neurons in the auditory cortex, leading to a gain increase [14]. Amplification of spontaneous activity then causes a synchronized activation that is above the perceptual threshold.

But why doesn’t the brain habituate to this irrelevant neural signal that does not carry any information? Chronic tinnitus can also be seen as a persisting aversive memory network, that is still connected with the initial noise trauma or stress that is often reported by tinnitus patients [15]. Negative appraisal of tinnitus and negative attention might prevent the perceived tone to be interpreted as irrelevant.

**Fig 1:**
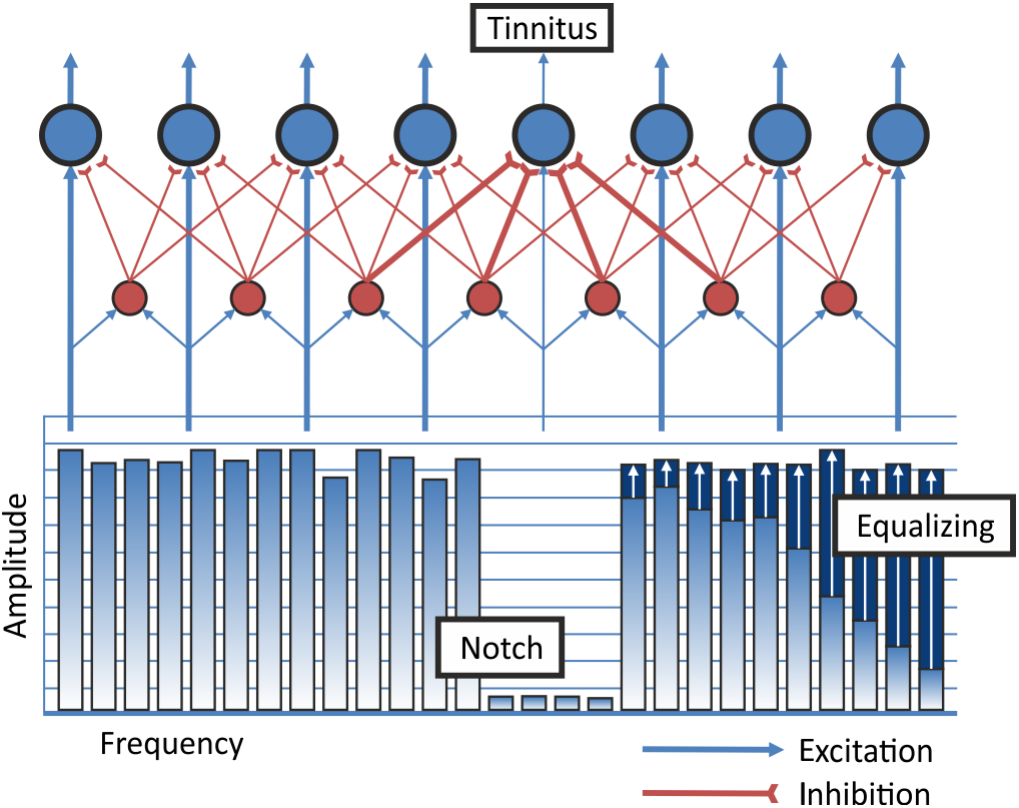
TMNMT Neurophysiological Mechanism and Music Modification. The individual tinnitus frequency is removed from the frequency spectrum of the music. This leads to increased lateral inhibition onto the neurons inside of the notch. Blue circles indicate excitatory pyramidal cells in auditory cortex. Red circles indicate inhibitory interneurons. Redistribution of energy from low frequencies to high frequencies is labeled as equalizing of the energy spectrum of the music.

If the theory holds true that in tinnitus there is a lack of lateral inhibition in auditory cortices, a training that boosts lateral inhibition into the hyperactive neuronal network should help with tinnitus. One possible auditory stimulus to elicit lateral inhibition onto neurons coding specific frequencies is band-eliminated white noise. This notch-filtered noise reduces the evoked activity in the auditory cortex for sounds that fall into the region of the notch [9]. This mechanism was used to design a treatment strategy against tonal tinnitus, the tailor-made notched music training (TMNMT). Tinnitus patients listen to music from which the frequency spectrum of the tinnitus has been removed by means of a notch filter. The notched music spectrum is used to evoke lateral inhibition from neurons tuned to adjacent frequencies outside of the notch onto the neurons within the notch involved in the tinnitus percept. In the same time, due to the notch, those neurons do not get any excitatory input (cf. Fig 1). Twelve month of listening to music, from which the frequency spectrum of the tinnitus had been removed, led to a reduction of cortical activity measured by the N100-component (N1m) and the auditory steady state response (ASSR) related to the tinnitus pitch and to a reduction of subjective tinnitus loudness [16]. This effect was superior to a placebo effect. Positive effects for TMNMT on tinnitus were already found after a short and more intense training for patients with tinnitus frequencies below 8 kHz [17]. The size of the effect of lateral inhibition depends on the bandwidth of the notch that is used. While Okamoto et al. [16] used a 1-octave notch width, a notch width of ½ octave also proved to be effective after three days of exposure to TMNMT [18]. In a study with band-eliminated broad band noise and healthy participants, eliminated bandwidths of a ¼-octave and a ½-octave caused an even larger N1m decrement than notches of one whole octave in a forward masking paradigm [19]. However, there appears to exist a critical bandwidth, as 1/8-octave did not lead to any further enhancement of the lateral inhibition. The effect of stronger lateral inhibition with smaller notch widths can be explained with stronger connections of neurons closer to the center frequency of the notch, as the critical role of the edge frequencies of the notch has also been demonstrated [20]. So far, the effect of notch width has only been tested for a 1000 Hz stimulus and for forward masking paradigms in normal hearing subjects. It remains unclear, if the same effect will show for higher frequencies and after long-term training in tinnitus subjects.

In our study we tested whether the effectiveness of TMNMT could be enhanced by using notch widths narrower than one octave. Therefore, we compared in a double-blind study a group that listened to music with one-octave notch width with a group that listened to music with a ½-octave notch and a group that listened to music with a ¼-octave notch width. We hypothesized that TMNMT would improve tinnitus related distress measured before and after the training in all three groups (Hypothesis 1). For the tinnitus distress, we expected an interaction of time point (pre versus post) and treatment group (Hypothesis 2). Furthermore, we expected a linear trend for the three groups with strongest improvement of tinnitus related distress in the ¼–octave group and the weakest improvement in the one-octave group (Hypothesis 3). We also expected a reduction of the N1m component (Hypothesis 4) and the ASSR (Hypothesis 5) in response to the presentation of the tinnitus tone relative to a control tone. Parallel to the behavioral data, we expected an interaction between reduction in the neural components and the treatment group, which should resemble the findings of Okamoto et al. [19] (Hypothesis 6 & 7). Furthermore, we hypothesized that a reduction of distress would positively correlate with a reduction of the neurophysiological auditory evoked response (Hypothesis 8).

## Methods and Materials

### Participants

Participants were recruited in the same way as described by Pantev et al. [21]. Inclusion criterions were as follows: Participants with chronic (> 3 months) tonal tinnitus were included in the study. We considered a whistling or beeping sound as tonal, while the perception of a noise-like sound was considered as non-tonal. Furthermore, participants were only included when the pitch perception of their tinnitus was subjectively stable over time and below or equal to 8.5 kHz. No patients with ENT, neurological or psychiatric disorders were included. Hearing loss had to be equal or less than 50 dB HL for all frequencies between 125 Hz and 8.5 kHz and equal or less than 40 dB HL for the individual tinnitus frequency. We had to limit the value of hearing loss and tinnitus frequency within those boundaries for two reasons. First, we wanted to make sure that enough spectral energy of the music would be perceived in the frequency bandwidth under investigation. Second, to measure stable cortical auditory responses we wanted to present stimuli with 35 dB above hearing level. The specific auditory stimulation system of the magnetoencephalographic (MEG) laboratory limits the maximum presentation loudness, which is even more the case for frequencies above 8.5 kHz. A total number of 34 participants were included. The study was conducted according to the Declaration of Helsinki and the study protocol was approved by the ethics committee of the Medical Faculty of the University of Münster. Each participant signed an approved informed consent form prior to study enrollment.

### Study Design

**Fig 2:**
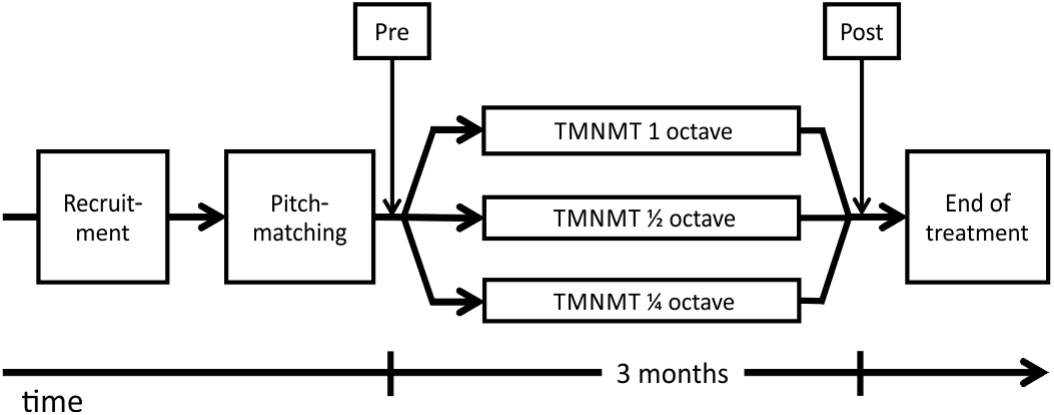
Flowchart of the Study Design. Participants were recruited and their tinnitus frequency matched before trial start. Following recruitment and tinnitus pitch-matching, participants received the pre measurement (behavioral and neurophysiological). After three month of TMNMT with 1-, ½- or ¼-octave notch width this was followed by the post measurement (same measures as pre).

After enrollment, participants received an iPod with Sennheiser HD201 headphones to perform a pitch-matching task at home over the following week. In a second session, two weeks later, after analyzing the pitch-matching data, we made a final decision whether the participant could be included into the study based on their tinnitus frequency. Included participants were scheduled for the pre-treatment MEG and behavioral data measurement (Fig 2). Four days after the baseline measurement, participants started with the TMNMT. Participants listened to tailor-made notched music for twelve weeks. Three days after the last training day, participants returned for the post measurement of behavioral and MEG data.

### Tinnitus Pitch Matching

To determine the tinnitus frequency, we used a combination of two different pitch-matching procedures. In the first procedure, participants rated (0–10) the similarity of their tinnitus to 37 pure tones ranging from 2 kHz to 16 kHz at home on their iPod over seven days [22]. Participants were instructed to use the Sennheiser HD201 headphones and adjust the loudness of each tone, so that it would match the individual tinnitus loudness. Subsequently, we calculated the mean ratings over the seven days. We then extracted the five peak ratings. Participants performed a forced-choice test with these frequencies to determine the final frequency. If the likeness rating curve did not provide those clear peaks, we additionally used a recursive two-interval forced-choice testing. This procedure features an algorithm to narrow the tinnitus frequency down by presenting frequencies from a low interval and a high interval [23,24]. The participants had to decide which of two tones was more similar to their tinnitus. Intervals were large in the beginning and became smaller with every decision until the final frequency was determined. An octave confusion test completed the testing. Participants performed two to three runs with this procedure. The median result from this procedure was then taken as the final tinnitus frequency estimate.

### TMNMT

Participants were asked to bring up to ten CDs of their favorite music collection to our institute. The music was transformed to high-resolution mp3 (320 kbit/s) format and copied to the participant’s iPod. The music was modified “on-line” with an application running on the iPod that has been developed for this purpose. Before the iPod was handed over to the participant, the notch width and the individual tinnitus frequency was set. The filtering of the music included equalizing of the music’s energy spectrum (c.f. Fig 1). This was achieved by redistribution of energy from low to high frequency ranges. This was supposed to guarantee an equal amount of spectral power below and above the frequency area suppressed by the notch filter. According to the treatment group a frequency band of 1-octave, ½-octave or ¼-octave around the tinnitus frequency was removed from the spectral energy of the music. We instructed participants to listen to the filtered music, using the Sennheiser HD 201 headphones, two hours a day. Participants were allowed to perform any silent activities while listening to the music.

### Outcome measures

We measured tinnitus distress with the total score of the German version of the Tinnitus Handicap Questionnaire (THQ) as the primary behavioral outcome measure [25]. As recommended by Landgrebe et al. [26]we also administered the Tinnitus Handicap Inventory (THI; [27]) and the Tinnitus Questionnaire (TQ; [28]). To measure subjective tinnitus loudness we used an iPod-based visual analog scale (VAS). We asked participants whether their tinnitus had improved, stayed the same or got better on a clinical global impression scale (CGI). In order to test whether the different notch widths influenced the perceived quality of the music we asked participants to rate the level of music enjoyment and how well they were able to relax while listening on a VAS (0–100).

Furthermore, we recorded neurophysiological data by means of MEG. Our goal was to trace back neuroplasticity effects on tinnitus elicited by TMNMT. Specifically, we used the auditory evoked N1m component and the ASSR. The MEG measurements were conducted in a magnetically shielded and acoustically silent room by means of a 275 channel whole-head MEG system (OMEGA 2005 WC, CTF Systems Inc., Port Coquitlam, Canada) equipped with axial gradiometer configuration of the pickup coils. The magnetic field signal was digitally sampled at a rate of 600 Hz. The measurement started with a recording of five minutes resting state with no task and no visual input (blank screen). Subsequently, we recorded the above mentioned auditory evoked fields. We presented two different sound stimuli, the individual tinnitus frequency (TF) and a control frequency (CF) of 500 Hz, to the participant in a randomized order. The stimuli were presented randomly either to the left or the right ear. Stimuli lasted for 1000 milliseconds (ms). The initial 300 ms were a pure sine wave followed by 700 ms of 40 Hz amplitude-modulated (100% modulation depth) sine wave with the same carrier frequency. This stimulus configuration allowed simultaneous recording of both, N1m and ASSR [29]. All stimuli had a 20 ms rise and fall time. The stimulus onset asynchrony was randomized between 2 and 3 seconds. 200 trials were presented in four runs with a short break in between each run. Each run thus resulted in 200 trials per condition (TF left, TF right, CF left, CF right). Prior to each measurement, we determined hearing thresholds for the CF. The CF was then presented with 35 dB SL (above sensation level). The loudness of the TF was matched to the CF before the baseline measurement. The loudness difference was kept identical for the post measurement. In order to keep the subject in an alert state, we presented a silent movie during the recordings.

### Randomization and Blinding

The allocation of a subject to one of the three groups was designed to minimize group differences. The criteria we aimed to match were age, tinnitus pitch, hearing loss, THQ score, Allgemeine Depressionsskala Langform (ADS-L) score and time since tinnitus onset. We used an F-Statistic to calculate the significance of group differences for these variables. Neither the participant, nor the investigator interacting with the participant knew at any time point to which group the participant was allocated. Participants were told that there were three different TMNMT groups and one placebo group, even though there was none. This should ensure that the placebo effect would be more comparable with the study of Okamoto [16] who also used a placebo group. We asked participants after they had completed the training to guess in which group they had been included. Participants were informed about their group allocation after the study was completed. Additionally every participant received a copy of his or her individual music modified in the way described above using the notch width that most participants reported to reduce tinnitus loudness.

### Statistical Analysis and MEG Preprocessing

To test our first three hypotheses, we calculated a 2 x 3 repeated measures mixed model ANOVA with time (pre and post) as the within subjects factor and group (1-octave, ½-octave, ¼-octave) as the between subject factor for the THQ score. Likewise, this was done with the scores of THI and TQ. A χ^2^ test was used to test whether the three groups differed in their responses on the CGI.

In order to analyze the MEG data, we initially segmented the data into epochs including the intervals from 350 ms pre to 1200 ms after stimulus onset. The data were filtered with a 30 Hz digital low-pass filter and baseline-corrected by subtracting the magnetic field signal averaged across the pre-stimulus interval from -300 ms to -50 ms. Channel wise, field changes larger than 2.5 pT were considered as artifacts and affected trials were excluded from further analysis. A complete channel was rejected, if more than 25% of the trials were contaminated by artifacts. Subsequently, we averaged the data for all four conditions (CF left, CF right, TF left, TF right) and also calculated a grand average of those. We then used the grand average data to perform a source analysis on the measured field distribution of N1m responses, applying a single equivalent current dipole (ECD) for each hemisphere. The N1m response latency was determined by the maximum of the global field power across all channels in the time interval from 0.085 to 0.145 s. Derived from that, we used a time window of 0.016 s prior to the peak on the rising slope of the N1m response for the spatiotemporal dipole fit in each hemisphere. Dipole location and orientation were calculated in a head-based Cartesian coordinate system with origin at the midpoint of the medial-lateral axis (y-axis) between the entrances of the left and right ear canals. The x-axis was defined as the posterior-anterior axis and the z-axis was orthogonal to the x-y plane. We accepted ECD fits with a goodness of fit of at least 90% and with at least 3 cm distance from the mid-sagittal plane. Via source space projection, this ECD template was used to calculate source wave forms for each condition and each hemisphere [30]. The N1m peak amplitude of the source waveform between 0.07 and 0.15 s after stimulus onset was used for the statistical analysis. In order to examine differences in the dipole fit between pre and post session we calculated the distance between the dipole locations and the angle between orientation of pre and post measurements.

For the ASSR analysis the averaged data were band-pass filtered between 32 and 48 Hz. The time interval of 0.5 to 1.0 s of the grand average was used for a complex demodulation to extract the 40 Hz component of the signal. The template model was derived from an ECD fit applied to the field distribution at the latency of the global field maximum of two periods of the demodulated signal. We accepted ECD fits that were located at least 2 cm distant from the mid-sagittal plane. The maximum source strength of ASSR for each condition was again calculated by means of source space projection. The envelope of these source waveforms was then calculated using a Hilbert transformation. For the statistical analysis of the ASSR we used the mean envelope in the time window from 0.5 to 1 s after stimulus-onset [9,31].

To control for effects of head position differences within subjects between pre and post measurement, we calculated ratios between source strengths evoked by the tinnitus frequency vs. the control frequency. To allow a comparison between subjects, we normalized the post data relative to the baseline data, and then calculated the changes of the normalized ratios {[(source strength elicited by tinnitus frequency at post / source strength elicited by control frequency at post) / (source strength elicited by tinnitus frequency at baseline / source strength elicited by control frequency at baseline) − 1] × 100} [16]. By means of a t-test, we tested whether the change score differed between hemispheres and calculated the mean of the left hemisphere and the right hemisphere. The overall effect of an N1m source strength decrement was tested via a t-test of the change score against zero. The change scores were also entered into an ANOVA with one within factor notch width condition. In order to test the last hypothesis, we calculated the correlation between the change values of the behavioral measures and the change in the neurophysiological responses.

## Results

28 of the 34 participants completed the 12 weeks of training. Reasons for dropout were: tinnitus got louder (n = 2), training was too time-consuming (n = 3), no reason given (n = 1). Two dropouts were in the 1-octave group, one in the ½-octave group and two in the ¼-octave group. Nevertheless, all but two participants were willing to complete the post measurement. We decided to use all available data for the analysis in order not to lose the information provided by the dropouts. In a supplementary analysis, we analyzed only the 28 participants who completed 12 weeks of training with TMNM. The main results of this analysis can be found in S1 Text. As described above, we were able to include 32 participants in the analysis of the behavioral measures with dropouts included. The baseline data of these 32 participants is shown in Table 1. None of these variables differed significantly between groups at baseline. In the one-octave group 25% of the participants guessed their group correctly, in the ½-octave group it were 20% and in the ¼-octave group no participant guessed correctly.

**Table 1:** Comparison of baseline data.

	1 octave	½ octave	¼ octave
**N**	12	10	10
**Gender (female)**	4	3	4
**Age (years)**	44.52 (11.27)	44.31 (11.56)	47.02 (5.51)
**Tinnitus pitch (Hz)**	4825 (917)	5577 (769)	6160 (661)
**Tinnitus duration (years)**	10.26 (7.43)	8.99 (6.45)	8.19 (7.31)
**Hearing loss (dB HL)**	16.29 (10.08)	16.84 (13.70)	17.29 (9.36)
**ADS-L**	8.00 (4.49)	5.90 (2.47)	8.60 (4.79)
**THQ**	21.82 (15.08)	22.88 (7.99)	21.34 (9.74)
**THI**	23.33 (14.76)	20.40 (9.13)	22.80 (9.58)
**TQ**	21.75 (12.68)	24.40 (9.73)	19.50 (8.96)

**Fig 3:**
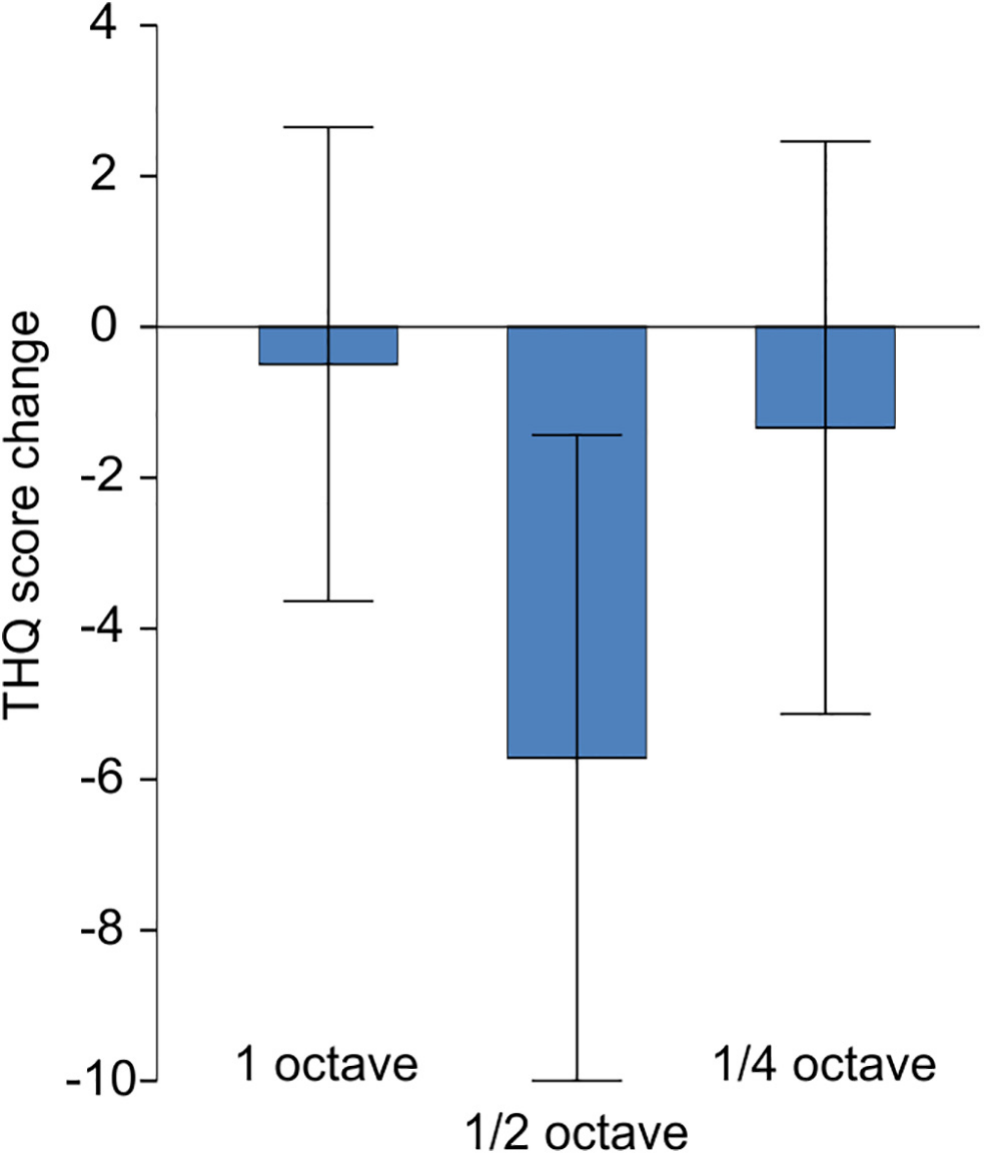
Behavioral Results. Mean change of THQ score in the three notch width groups. Error bars indicate 95%-CI. A negative change indicates an improvement of tinnitus distress.

Overall, patients improved in their THQ scores from pre to post, as the main effect for time of the 2 x 3 repeated measures ANOVA became significant, *F*(1,29) = 6.899; *p* = 0.014; *Eta* ^2^ = .192. The interaction of group * time point did not reach significance, *F*(2,29) = 2.829; *p* = 0.075; *Eta* ^2^ = .163 (Fig 3). The mean reduction of tinnitus distress was 11% (2 to 20; 95% CI). For the THI, we observed a similar result: Scores were lower at the post measurement, *F*(1,29) = 6.960; *p* = 0.013; *Eta* ^2^ = .194. The interaction group x time point was not significant, *F*(2,29) = 1.747; *p* = 0.192. Scores of the TQ also showed the effect for time, *F*(1,29) = 4.323; *p* = 0.049; *Eta* ^2^ = .127, but not for time * group, *F*(2,29) = 0.832; *p* = 0.445. The change scores of the three questionnaires were also significantly correlated, THQ and THI, r = 0.551; *p* < 0.01, THQ and TQ, r = 0.581; *p* < 0.01, THI and TQ, r = 0.732; *p* < 0.01. Due to over 15% of missing data, we did not include the results from the iPod based subjective loudness measure in our analysis.

In the 1-octave group, 1 participant stated that the tinnitus had become worse, 10 answered that their tinnitus had not changed and 1 had the impression that the tinnitus had become better. For the ½-octave group the distribution was: 1 got worse, 4 no change, 5 improved and for the ¼-octave group 1 got worse, 9 no change and 0 improved. The χ^2^ test revealed that this difference in the distribution of the CGI was different across groups, χ^2^(4) = 9.890; *p* = 0.042. The level of music enjoyment did not differ between the three groups, *F*(25,2) = 0.194, *p* = 0.825, neither did the level of relaxation, *F*(25,2) = 0,745; *p* = 0.485.

**Fig 4:**
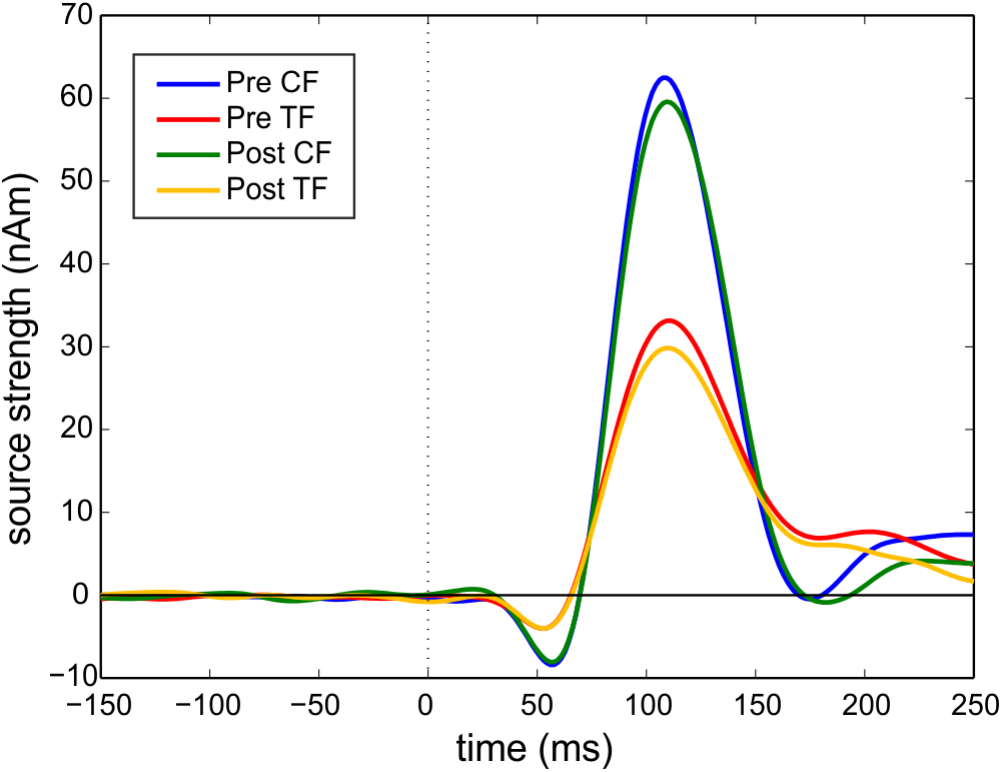
Grand averaged source waveforms for the N1m time window. Pre CF = pre measurement control tone. Pre TF = pre measurement tinnitus tone. Post CF = post measurement control tone. Post TF = post measurement tinnitus tone. The onset of the stimulus is at 0 milliseconds and indicated by the dotted line. N1m source strength is lower for the TF, because the TF has a higher carrier frequency than the CF.

We excluded six participants from the analysis of MEG data because of the following reasons: Two participants had a head circumference that was too large to fit into the MEG dewar. One participant showed more than 25% artifacts (field changes larger than 2.5 pT) in 109 (pre) and 128 (post) channels. One participant was an outlier due to the N1m response peak latency, which was determined at 0.160 s. The fitted dipolar sources of one participant were localized clearly outside of the auditory cortex region. The absolute source strengths of one participant were more than two standard deviations higher than the mean of the other participants. The same participant was also an outlier concerning deviation of the dipolar sources from pre to post measurement (left: 2.2 cm; right: 2.2 cm) as well as orientation deviation (left: 39°; right: 31°). The mean goodness of fit of the remaining 26 participants was 0.963 (SD = 0.022) for pre measurement and 0.967 (SD = 0.021) for post measurement. Examination of the contour maps revealed clear dipolar field pattern. Grand averaged source waveforms for the N1m time window are shown in Fig 4 for the mean of all groups and in S1 Fig for each group separately. The mean Euclidean distance of the N1m dipole locations between pre and post measurement was 0.64 cm (SD = 0.39 cm) for the left dipolar source and 0.65 cm (SD = 0.38 cm) for the right dipolar source. However, neither for x-, y- or z-coordinate nor for left and right hemisphere this spatial difference was significant. The mean angle between dipole orientations was 6.73° (SD = 5.56°) for the left and 5.98° (SD = 4.23°) for the right hemisphere.

**Fig 5:**
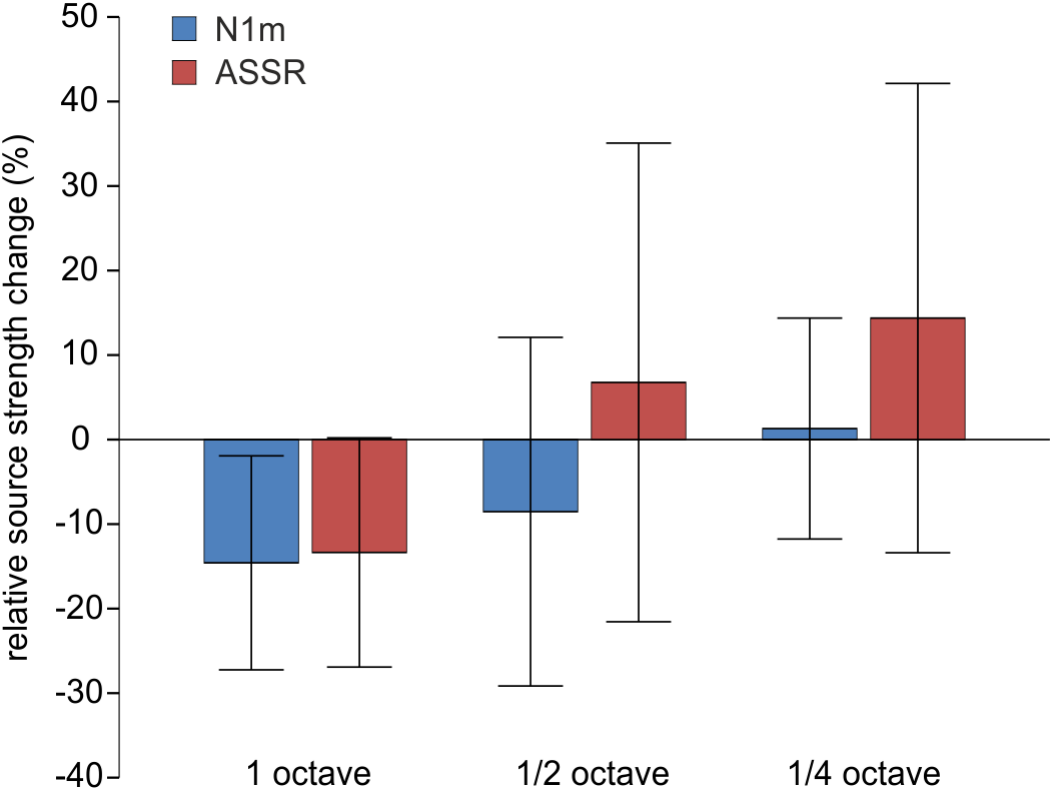
Neurophysiological Results. Mean relative change of N1m (blue) and ASSR (red) source strength evoked by the tinnitus tone relative to baseline and a 500 Hz control tone. Error bars indicate 95%-CI.

The baseline source strength values of the N1m did not differ between groups in the MANOVA with the factor group and the dependent variables pre TF left, pre TF right, pre CF left and pre CF right, F(8,40) = 0.373, *p* = 0.928; Wilk's Λ = 0.866. The change scores for N1m source strength did not differ between hemispheres, t(25) = 0.391; *p* = 0.699, therefore we used the mean of left and right hemisphere for the following analysis. The training induced a lasting effect of inhibition, as the overall change score tested for all subjects was negative, t(25) = -1.871; *p* = 0.037. However, there was no significant effect of the notch width in the ANOVA, F(2,23) = 1.290; *p* = 0.295. Fig 5 illustrates the relative change of the N1m source strength.

For the analysis of the ASSR, the data of four additional participants had to be excluded from this analysis because the ECD fits resulted in locations clearly outside of the auditory cortex. The data of 22 participants was used to analyze ASSR source strength changes. The baseline source strength values of the ASSR did also not differ between groups in the MANOVA with the factor group and the dependent variables pre TF left, pre TF right, pre CF left and pre CF right, F(8,32) = 0.884, *p* = 0.541; Wilk's Λ = 0.671. As for the N1m source strength, the change scores did not differ for hemispheres, t(26) = 0.031; *p* = 0.976, therefore we used the mean of left and right hemisphere for the following analysis. For the ASSR, we did not find a lasting effect of inhibition, t(21) = 0.168; *p* = 0.444. Although notch width appeared to have an effect on the change scores of ASSR source strength, this difference did not reach significance F(2,19) = 2.723; *p* = 0.091. The change scores of the ASSR are also shown in Fig 5.

For both N1m source strength change and ASSR source strength change we calculated the Pearson product-moment correlation coefficients with the change in the THQ, THI and TQ. THQ change did neither correlate with change in the N1m source strength, r = 0.08; *p* = 0.725, nor with ASSR source strength, r = -0.151; *p* = 0.502. All correlations with the other questionnaires did also not reach higher levels of significance.

## Discussion

In line with our first hypothesis, three month of listening to TMNM led to a significant reduction of tinnitus distress. Thereby, our results are consistent with the interpretation that TMNMT enhances lateral inhibition from frequencies adjacent to the tinnitus frequency, which then leads to a decrease of neural activity [16,17]. The magnitude of the neurophysiological effect seems to be somewhat lower than in the twelve month lasting study, which can be explained by the shorter duration of the training. The magnitude of the behavioral effect is difficult to compare with previous studies, as in those studies a wide range of outcome measures has been used. Despite the statistical significance, the behavioral effect has to be considered as below clinical significance. Newman et al. [32] reported 21 points of reduction to be necessary for minimally clinically important difference in the THQ. The small effect can be explained by the generally low distress level of the participants. High levels of tinnitus distress are often accompanied by mental disorders [33]. Through excluding tinnitus patients with psychiatric comorbidities, we might have restricted our sample to individuals with only mild distress levels. Thus, this could have led to a floor effect, where a large improvement was unlikely.

A major goal of the study was to identify the notch width that would provide the best training effect. We expected the ½-octave condition to be superior to the 1-octave condition and even the best outcome for the ¼-octave condition. Yet, this pattern did not reflect in our results. Although the results of the THQ and the other distress measures did not reveal a clear effect of notch width, more participants reported an improvement on the CGI scale in the ½-octave group. This might be interpreted as a hint that the ½-octave notch width can be regarded as most promising to induce an improvement in tinnitus patients.

The above mentioned floor effect could be responsible for this. Another explanation for the missing effect of notch width in the standardized outcome measures could be that a narrow notch might not continuously cover the tinnitus frequency over the training time period. Tinnitus subjects differ largely in the variability of their pitch-matching [34]. The pitch-matching procedure, used in this study did not allow us to measure the reliability of our participants. But even with a reliable estimation of the tinnitus pitch, it is feasible that the tinnitus percept itself is broader than the notch widths under investigation. Some participants reported that their tinnitus does not sound like a pure tone and the literature supports the idea that an individual frequency spectrum rather than a single tinnitus frequency might be more suitable to describe the tonal tinnitus percept [22]. This would mean that narrowing the notch for TMNMT might increase the probability that the notch misses the actual tinnitus frequency or frequency spectrum. One option to compensate for that in further studies is to estimate the corresponding confidence interval based on the variability of the individual participant’s pitch-matches. Those confidence intervals should then be used to determine the optimal notch width for the TMNMT.

On the neurophysiological level we were able to confirm the results of previous studies on lateral inhibition (cf. [9,31]). The N1m source strength of the evoked responses was reduced for the tinnitus pitch relative to the control tone over all three groups. This general effect of lateral inhibition was even present three days after the training has been completed. In most previous studies, the time difference between the end of the training and the MEG measurement was shorter. Yet, in one study, the neurophysiological effect was also significant after three days but not shortly after TMNMT [17]. We expect the effect of lateral inhibition to decay over time. Nevertheless, we were able to show that it is stable at least for a couple of days. However, we found the relative reduction of neurophysiological source strength only for the N1m response that originates in the secondary auditory cortex and not for the ASSR that represents earlier stages of auditory processing. This finding is in line with other studies investigating lateral inhibition: Pantev et al. [9] argued that the hierarchical structure of the auditory system is responsible for this differential effect. Stein et al. [20] also found the effect caused by notch filtered sound only for the N1m response and not for the ASSR.

As for the behavioral measures, in this three months training study we did not find an effect of notch width on the reduction of the N1m cortical source strength. Thus, we were not able to confirm our hypothesis that notch widths narrower than one octave are more efficient in inducing lateral inhibition in tonal type of tinnitus. How can we explain this contrast to the study of Okamoto et al. [19]? In their study band-eliminated broad band noise was used to inhibit the neural response to a 1 kHz test tone in a short-term forward masking paradigm. This masking paradigm is eliciting short lasting lateral inhibition, whereas the TMNMT paradigm is designed to generate long lasting effects. Therefore, different underlying neural mechanisms could to certain extent explain the differences.

Another important difference is the investigated sample. While Okamoto et al. [19] used healthy participants with normal hearing; we used tinnitus patients with hearing loss in the current study. As it has been suggested, lateral inhibition could be generally weaker in tinnitus patients and also can be influenced by the existing hearing loss [12,35]. Okamoto et al. [19] found that the lateral inhibition effect was strongest at ¼-octave notch width. However, it is possible that the function between notch width and lateral inhibition is not linear over the whole frequency range. The cochlea is most sensitive approximately between 2 and 5 kHz. As there are differences in excitability, there could also be differences in the range of lateral inhibition for different frequencies. Furthermore, neurons coding the notch frequency could be activated by the edge frequencies of a narrow notch, because of broader excitatory lateral connections in higher frequencies. This would then prevent the inhibitory effect of lateral inhibition. As the tinnitus frequency was above 5 kHz for more than 50% of the participants, this could explain, why notches narrower than one octave did not lead to a more pronounced effect of lateral inhibition. However, it would be necessary to carry out studies that use a more specific design to address a possible asynchrony of lateral inhibition in high frequencies.

Another reason, why the group differences in our study did not reach statistical significance might be the lack of statistical power. Substantial number of participants had to be left out in the analysis of MEG data. As the study took several month and some participants had to be excluded due to their post data, it was not possible to replace them. Furthermore the stimulation level we have used was 10 dB less than the stimulation level used in the Okamoto et al. study [16,19]. However, it is obvious that this reduction of the stimulation level decreased the signal-to-noise-ratio of the recorded N1 and ASSR evoked responses. The problem is the trade-off between hearing threshold and frequency dependent limitations of the MEG stimulation systems that cannot be always overcome in the case of comparative studies between normal hearing subjects and tinnitus patients with hearing loss.

With respect to the correlation between the ASSR and subjective tinnitus loudness demonstrated by Okamoto et al. [16], that we were not able to confirm in the current study, we would like to comment that it seems that this correlation is not very consistent. Other studies were also not successful to replicate it [17,36] or reported a correlation between change of tinnitus loudness and N1m decrement [18]. Besides the addressed methodological problems, there are more general reasons why comparing subjective ratings with neurophysiological data can be intricate. Subjective measures always contain a retrospective evaluation. In most items of the tinnitus questionnaires, participants are asked to think of situations they have experienced in the past that are in some way influenced by the tinnitus. Furthermore, a person with tinnitus has experience with different loudness levels and will compare the actual loudness with all these remembered levels. In contrast, neurophysiological measures—like MEG—determine the activity of the brain in well-defined short time frames. The paradigm used in this study was designed to measure activity that is believed to correlate with the activity underlying tinnitus. However, up to now there is no univocal evidence that this link between neural activity and the behavioral percept clearly exists.

For this study several changes were made to the original TMNMT. One major difference was the duration of the training: 3 months in the current study versus 12 months in the study of Okamoto et al. [16]. However, they found a significant reduction of tinnitus loudness and source strength already after 6 month of TMNMT relative to baseline but not superior to the placebo group.

A general limitation of the study seems to be the lack of an a priori power analysis. The performance of MEG measurements and the long duration of the study limited our number of participants. Furthermore, data from previous studies was not sufficient to calculate a power analysis. Due to the lack of a direct effect of notch width on the behavioral data, it is also possible that the overall positive effect of the training is strongly influenced by an unspecific placebo effect or a regression to the mean. A control group design would be helpful to rule this out.

In summary, in the current experiment we were able to show that listening to notched music leads to a reduction of neural activation in the notched frequency area that was still observed three days after TMNMT offset. After three month of TMNMT tinnitus distress was significantly reduced, although this change was not clinically significant. However, we were not able to find an effect of notch width neither on the tinnitus distress nor on the evoked electrophysiological auditory cortex activity. Nevertheless, in the ½-octave group the most participants reported that the training had improved their tinnitus. However, the only placebo controlled study with TMNMT has been done with the 1-octave notch width, so far [16]. Considering these results and our own results, we recommend to consider 1-octave and further evaluate the ½-octave condition but not to use a notch width narrower than this.

## Supporting Information

S1 Fig Grand averaged source waveforms for the N1m time window separated by group. Pre CF = pre measurement control tone. Pre TF = pre measurement tinnitus tone. Post CF = post measurement control tone. Post TF = post measurement tinnitus tone. The onset of the stimulus is at 0 milliseconds and indicated by the dotted line. N1m source strength is lower for the TF, because the TF has a higher carrier frequency than the CF.
(TIF)

S1 File Complete Dataset. Dataset containing all data analyzed and presented in the current study.
(SAV)

S1 Text Supplementary analysis with all participants who completed 12 weeks of TMNMT (n = 28). The main analyses reported in the results section were recalculated for this reduced sample.
(DOCX)
