# Supplementary figures and images for: Impact of Spectral Notch Width on Neurophysiological Plasticity and Clinical Effectiveness of the Tailor-Made Notched Music Training
Source: PLoS One. 2015 Sep 25;10(9):e0138595. doi: 10.1371/journal.pone.0138595 (PMC4583393; doi:10.1371/journal.pone.0138595)

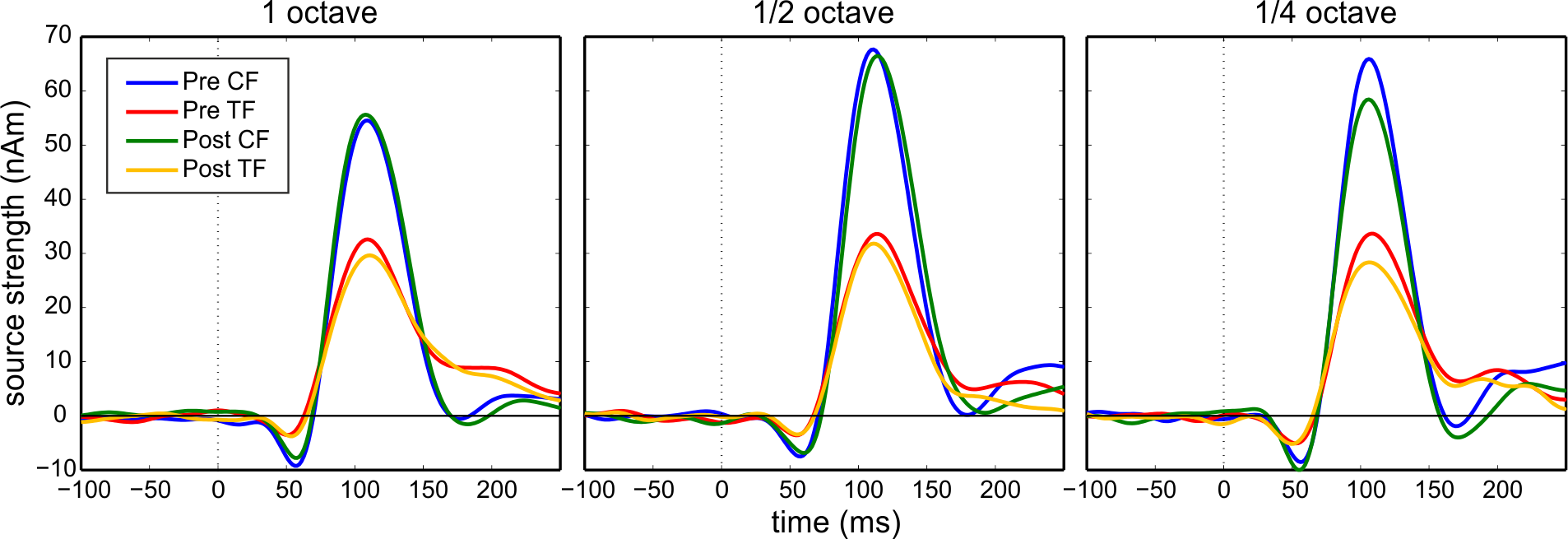

Supplement: S1 Fig — Pre CF = pre measurement control tone. Pre TF = pre measurement tinnitus tone. Post CF = post measurement control tone. Post TF = post measurement tinnitus tone. The onset of the stimulus is at 0 milliseconds and indicated by the dotted line. N1m source strength is lower for the TF, because the TF has a higher carrier frequency than the CF. (TIF) [file pone.0138595.s001.tif]
