# Supplementary material for: Impact of Spectral Notch Width on Neurophysiological Plasticity and Clinical Effectiveness of the Tailor-Made Notched Music Training
Source: PLoS One. 2015 Sep 25;10(9):e0138595. doi: 10.1371/journal.pone.0138595 (PMC4583393; doi:10.1371/journal.pone.0138595)
Supplement: S1 Text — The main analyses reported in the results section were recalculated for this reduced sample. (DOCX) [file pone.0138595.s003.docx]

**Supplementary Analysis**

From the 28 participants who completed 12 weeks of training with TMNM, 10 were in the 1-octave group, nine in the ½-octave group and nine in the ¼-octave group. The three groups did not differ significantly in their baseline data.

Overall, patients improved in their THQ scores from pre to post, as the main effect for time of the 2 x 3 repeated measures ANOVA became significant, *F*(1,25) = 6.247; *p* = 0.019; 𝐸𝑡𝑎^2^= .200. The interaction of group * time point did not reach significance, *F*(2,25) = 1.861; *p* = 0.176; 𝐸𝑡𝑎^2^= .130. For the THI, we observed a similar result: Scores were lower at the post measurement, *F*(1,25) = 6.901; *p* = 0.015; 𝐸𝑡𝑎^2^= .216. The interaction group x time point was not significant, *F*(2,25) = 1.165; *p* = 0.328. Scores of the TQ did not show the effect for time, *F*(1,25) = 3.764; *p* = 0.064; 𝐸𝑡𝑎^2^= .131, and also not for time * group, *F*(2,25) = 0.732; *p* = 0.491.

We excluded five additional participants from the analysis of MEG data because of the following reasons: Two participants had a head circumference that was too large to fit into the MEG dewar. One participant was an outlier due to the N1m response peak latency, which was determined at 0.160 s. The fitted dipolar sources of one participant were localized clearly outside of the auditory cortex region. The absolute source strengths of one participant were more than two standard deviations higher than the mean of the other participants. The same participant was also an outlier concerning deviation of the dipolar sources from pre to post measurement (left: 2.2 cm; right: 2.2 cm) as well as orientation deviation (left: 39°; right: 31°). The mean goodness of fit of the remaining 23 participants (1-octave: n = 8; ½-octave: n = 8; ¼-octave: n = 7) was 0.963 (SD = 0.023) for pre measurement and 0.967 (SD = 0.021) for post measurement. The mean Euclidean distance of the N1m dipole locations between pre and post measurement was 0.65 cm (SD = 0.42 cm) for the left dipolar source and 0.67 cm (SD = 0.40 cm) for the right dipolar source. However, neither for x-, y- or z-coordinate nor for left and right hemisphere this spatial difference was significant. The mean angle between dipole orientations was 6.89 ° (SD = 5.74 °) for the left and 6.35 ° (SD = 4.33 °) for the right hemisphere.

The baseline source strength values of the N1m did not differ between groups in the MANOVA with the factor group and the dependent variables pre TF left, pre TF right, pre CF left and pre CF right, F(8,34) = 0.331, *p* = 0.948; Wilk's Λ = 0.861. The change scores for N1m source strength did not differ between hemispheres, t(22) = 0.296; *p* = 0.770, therefore we used the mean of left and right hemisphere for the following analysis. With this reduced sample, the effect of inhibition on source strength did not reach significance, as the overall change score tested for all subjects did not fall below zero, t(25) = -1.648; *p* = 0.057. There was also no significant effect of the notch width in the ANOVA, F(2,20) = 1.014; *p* = 0.381.

For the analysis of the ASSR, the data of three additional participants had to be excluded from this analysis because the ECD fits resulted in locations clearly outside of the auditory cortex. The data of 20 participants was used to analyze ASSR source strength changes. The baseline source strength values of the ASSR did also not differ between groups in the MANOVA with the factor group and the dependent variables pre TF left, pre TF right, pre CF left and pre CF right, F(8,28) = 1.014, *p* = 0.448; Wilk's Λ = 0.601. As for the N1m source strength, the change scores did not differ for hemispheres, t(19) = 0.214; *p* = 0.833, therefore we used the mean of left and right hemisphere for the following analysis. For the ASSR, we also did not find a lasting effect of inhibition, t(19) = 0.489; *p* = 0.630. Notch width did not influence ASSR source strength F(2,17) = 1.823; *p* = 0.192.
